# Associations of Drug Lipophilicity and Extent of Metabolism with Drug-Induced Liver Injury

**DOI:** 10.3390/ijms18071335

**Published:** 2017-06-22

**Authors:** Kristin McEuen, Jürgen Borlak, Weida Tong, Minjun Chen

**Affiliations:** 1 Division of Bioinformatics and Biostatistics, National Center for Toxicological Research, US Food and Drug Administration, Jefferson, AR 72079, USA; Kristin.McEuen@fda.hhs.gov (K.M.); weida.tong@fda.hhs.gov (W.T.); 2 Department of Information Science, University of Arkansas at Little Rock, Little Rock, AR 72204, USA; 3 Center of Pharmacology and Toxicology, Hannover Medical School, Hannover 30625, Germany; Borlak.Juergen@mh-hannover.de

**Keywords:** hepatotoxicity, drug dose, drug lipophilicity, metabolism, risk factor, annotation

## Abstract

Drug-induced liver injury (DILI), although rare, is a frequent cause of adverse drug reactions resulting in warnings and withdrawals of numerous medications. Despite the research community’s best efforts, current testing strategies aimed at identifying hepatotoxic drugs prior to human trials are not sufficiently powered to predict the complex mechanisms leading to DILI. In our previous studies, we demonstrated lipophilicity and dose to be associated with increased DILI risk and, and in our latest work, we factored reactive metabolites into the algorithm to predict DILI. Given the inconsistency in determining the potential for drugs to cause DILI, the present study comprehensively assesses the relationship between DILI risk and lipophilicity and the extent of metabolism using a large published dataset of 1036 Food and Drug Administration (FDA)-approved drugs by considering five independent DILI annotations. We found that lipophilicity and the extent of metabolism alone were associated with increased risk for DILI. Moreover, when analyzed in combination with high daily dose (≥100 mg), lipophilicity was statistically significantly associated with the risk of DILI across all datasets (*p* < 0.05). Similarly, the combination of extensive hepatic metabolism (≥50%) and high daily dose (≥100 mg) was also strongly associated with an increased risk of DILI among all datasets analyzed (*p* < 0.05). Our results suggest that both lipophilicity and the extent of hepatic metabolism can be considered important risk factors for DILI in humans, and that this relationship to DILI risk is much stronger when considered in combination with dose. The proposed paradigm allows the convergence of different published annotations to a more uniform assessment.

## 1. Introduction

Drug-induced liver injury (DILI) can result in severe clinical outcomes such as acute liver failure, and, although rare, DILI is encountered frequently during the drug development process and therefore presents a significant challenge to drug developers and regulators. Importantly, the complexity of the disease, the lack of predictive biomarkers as well as the difficulty to diagnose and determine causality are obstacles that need to be overcome [1,2,3]. Our current understanding of DILI pathogenesis is limited, and therefore identifying risk factors is critical for a better understanding and avoidance of DILI risks.

An increased risk of developing DILI is thought to be caused by interactions among the host, drug, and environmental factors [3,4]. Genetic studies have found single nucleotide polymorphisms in a number of genes, including human leukocyte antigen (HLA) regions that were associated with increased risk for DILI as shown for flucloxacillin, amoxicillin-clavulanate, and lapatinib [5,6,7,8,9]. Non-genetic host factors, such as age, gender, and underlying liver disease, were also found to be associated with increased DILI risk; however, many of these factors may be associated only with specific drugs [10]. Underlying liver diseases affect the activities of certain CYP enzymes such that drugs that are substrates of these enzymes should be given at lower doses [11]. Furthermore, there is evidence for serum acute phase reactants to hallmark healthy individuals at risk to develop DILI prior to drug treatment, therefore carrying the potential to identify individuals likely to develop DILI [12].

When considering drug properties that may have influence on DILI, lipophilicity is an important criterion that strongly contributes to the distribution of drugs throughout the body and is generally associated with a large volume of distribution. There is also increasing interest in investigating drug characteristics and their relationship to DILI risk. Lammert et al. found that exposure to oral medications given at doses greater than 50 mg/day was associated with a higher DILI risk [13]. This finding was later confirmed in the Spanish DILI registry, where 77% of the drugs that caused DILI were given at doses of 50 mg/day or more [14]. In addition to dose, extensive metabolism (>50%) is also associated with increased risk of hepatic adverse events from orally administered drugs [15]. Recently, we identified drugs with daily doses (DD) greater than 100 mg/day and high lipophilicity (measured by log value for octanol-water partition coefficient *p*, i.e., logP ≥ 3) to be associated with increased DILI risk [16]. These two drug characteristics (i.e., extent of metabolism and lipophilicity) look promising and have been widely cited by seminal reviews [3,10], but further validation to test the reliability of the findings in larger datasets is warranted.

In this study, we aim to comprehensively revisit the relationship between DILI risk and lipophilicity and the extent of metabolism using five independent annotations for drugs within the DILIrank dataset, a large published drug reference dataset with 1036 Food and Drug Administration FDA-approved drugs [17]. We found that lipophilicity and the extent of metabolism alone were associated with increased DILI risk, but that the strength of association varied considerably across different annotations. However, the combination of high daily dose with high lipophilicity or significant hepatic metabolism was consistently associated with increased DILI risk across all annotated datasets.

## 2. Results

**Figure 1:**
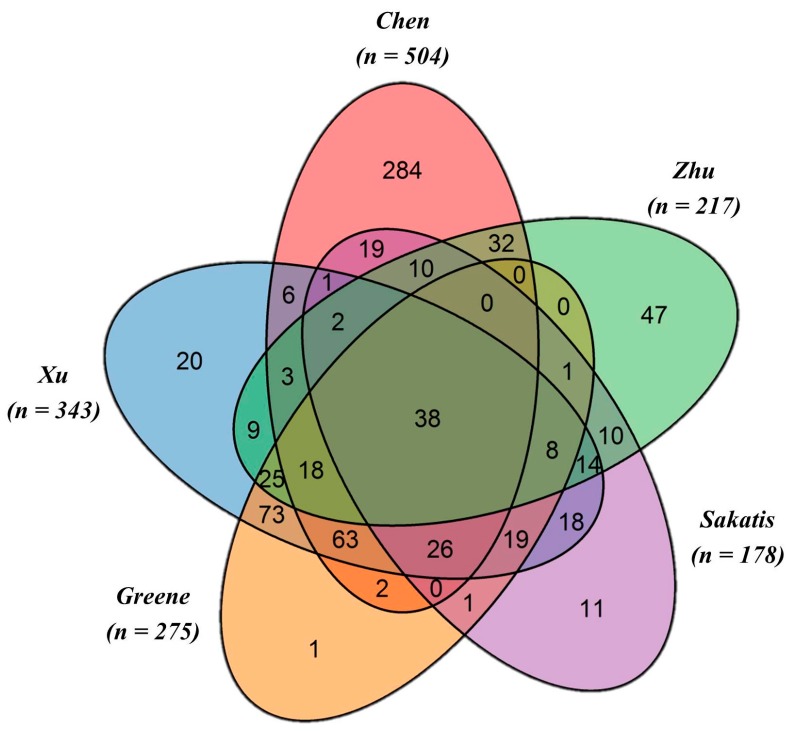
Venn diagram showing the commonality and specificities among the five individually annotated datasets. Among the different authors, a total of 38 drugs were commonly annotated. Highlighted in the Venn diagram are also specific data sets. For example, the Zhu et al. dataset contains *n* = 47 drugs uniquely annotated by these authors. Similarly, *n* = 11 unique drugs are considered in the Sakatis et al. data set. The R package VennDiagram [18] was used to generate this figure.

The Venn diagram (Figure 1) shows the commonality and the specificities of the DILI datasets retrieved from the five independent annotations for *n* = 1036 DILIrank drugs. A large portion of the drugs (*n* = 398) are annotated by at least two datasets, but only *n* = 38 drugs are in common among all five datasets. Chen et al. provides the largest annotation dataset of *n* = 504, with *n* = 192 ^v^Most-DILI-concern and *n* = 312 ^v^No-DILI-concern drugs, followed by Xu et al. of *n* = 343, with *n* = 195 DILI positives and *n* = 148 negatives, and Greene et al. of *n* = 275, with *n* = 189 drugs having human evidence of toxicity and *n* = 86 drugs with no evidence of human toxicity. Additionally, the Sakatis dataset annotated *n* = 178 drugs, including *n* = 92 hepatoxicants and *n* = 86 as non-hepatotoxicants, and the Zhu et al. dataset labeled *n* = 217 drugs, of which *n* = 161 were hepatotoxic and *n* = 56 were non-hepatotoxic. Notably, a total of *n* = 275 DILIrank drugs were not evaluated in the analysis due to their ambiguous annotations.

### 2.1. Lipophilicity and DILI Risk

We analyzed 763 medications retrieved from the DILIrank dataset with both daily dose and log*P* data available. To comprehensively assess the relationship between lipophilicity and hepatotoxicity risk, five independent DILI annotations were used, as detailed above. We used Fisher’s exact test to determine the statistical significance of the association between high daily dose, high lipophilicity (measured by log*P*), and DILI risk. The extent of the association was measured using odds ratio (OR) calculated by logistic regression.

As illustrated in Table 1, the combination of high lipophilicity (log*P* ≥ 3) and DD ≥ 100 mg, as defined by the Rule of Two (RO2) [16], is statistically significantly associated with the risk of DILI across all datasets (*p* < 0.05); however, the estimated OR ranges from 2.32 to 11.50, depending on the annotations used.

The association between DILI risk and high lipophilicity is statistically significant only when considering the annotations generated by Chen, Greene, and Zhu; however, no significant relationship was seen when the Sakatis or Xu annotations were considered. Note, DD ≥ 100 mg was significantly associated with DILI risk in all datasets. Furthermore, when the datasets of Chen, Greene and Zhu were evaluated, the OR for logP alone is significantly lower than those calculated for the combination of DD ≥ 100 mg and log*P* ≥ 3, suggesting a weaker association between log*P* alone with DILI risk as compared to the combination of DD ≥ 100 mg and log*P* ≥ 3.

**Figure 2:**
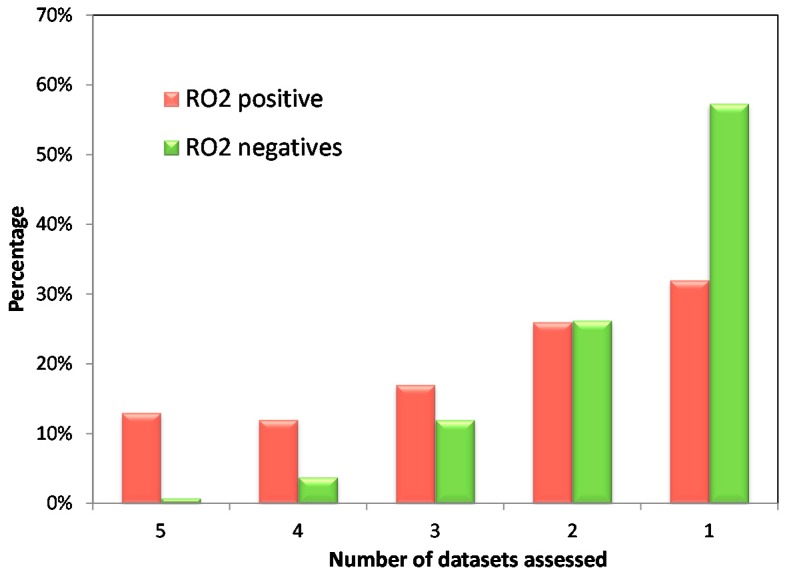
The overlap of Rule of Two (RO2) positives and negatives across the five investigated datasets. From the left to the right: Red bars depict drugs that are RO2 positives and the percentage of RO2 positives that were identified as hepatotoxic among all datasets (**left**) to only one dataset (**right**). Green bars depict drugs that are RO2 negatives and the percentage of RO2 negatives are in agreement with all five datasets (**left**) to only one dataset (**right**). To calculate the overlap of RO2 positives and negatives across the investigated datasets, a total of *n* = 763 drugs were considered, of which *n* = 139 were RO2 positives and *n* = 624 were RO2 negatives. The percentage of RO2 positives shared by all five datasets is much higher than the percentage of RO2 negatives. Importantly, the percent of RO2 negatives sharply decreases as the number of datasets increases, indicating a lack of consensus in the DILI classifications of RO2 negatives as compared to that of RO2 positives.

We also analyzed the RO2 results across the five annotated datasets. As shown in Figure 2, there were 13% of RO2 positives but <1% RO2 negatives which were shared by the five datasets. Similar trends were observed for the percentage of RO2 positives and negatives shared by three or four datasets. The higher consensus in RO2 positives than in RO2 negatives agrees with our previous report regarding the unique characteristics of the RO2 model, which has a high true positive rate but a high false negative rate [16].

### 2.2. Metabolism and DILI Risk

Similarly, a total of 559 DILIrank drugs with daily dose and available metabolism data were used to assess the relationship between DILI risk and the extent of metabolism. As given in Table 2, the combination of hepatic metabolism ≥50% and DD ≥ 100 mg was significantly associated with an increased risk of DILI across all datasets (*p* < 0.05), and the extent of association, as measured by ORs, ranged from 3.79 to 11.09 depending on the annotations used. Significant hepatic metabolism (≥50%) alone was significantly associated with DILI risk in most annotations except for the Zhu et al. and Sakatis et al. datasets. Similar to log*P*, the calculated ORs for the combination of metabolism and daily dose were higher than those for metabolism alone. This was seen in any of the five annotated datasets, which demonstrates that the combination with daily dose enhances the association between the extent of metabolism and DILI risk. As in the preceding analysis, DD ≥ 100 mg alone was significantly associated with DILI risk across all annotated datasets.

## 3. Discussion

The identification of DILI risk factors is of critical importance to protect individuals from adverse drug reactions. Recent seminal reviews include lipophilicity and the extent of metabolism as potential risk factors in the pathogenesis of DILI [3,10]. Given the inconsistencies among DILI annotations, it is important to further validate contributing factors to DILI onset and progression. In the present study, we utilized a large published DILIrank dataset with 1036 FDA-approved drugs and five independent DILI annotations to comprehensively assess the association between DILI risk and two drug properties, namely lipophilicity and the extent of metabolism. Based on a consensus approach, our results suggest that lipophilicity and the extent of metabolism alone had weak, but statistically significant, associations with an increase in DILI risk, and that factoring daily dose into the algorithm significantly strengthened these associations. 

In our analysis, we did observe significant variation across different annotations regarding the strength of the association between DILI risk and lipophilicity as well as the extent of metabolism. Obviously, the accuracy of annotating DILI risks varied among the datasets and this could affect the outcome when assessing risk factors. No golden standard for DILI annotation has been established so far, and each annotation utilizes different sources and criteria to define the risk of liver injury. For example, in the Xu et al. dataset, drugs with more than 10 clinical reports of Hy’s law cases are positive, while in the Sakatis et al. dataset, having 50 case reports of clinically significant hepatotoxicity was set as a threshold for drugs to be considered as DILI positive. Furthermore, to establish causality, the data needs to be evaluated by accepted methods for causality assessment such as RUCAM [24], though expert opinion is of equal importance [25]. Unfortunately, different outcomes among various causality assessment scales have been observed [26]; nonetheless, for some drugs the reported evidence of hepatotoxicity is vague [27,28]. Although not all of the datasets compared in the present study considered the RUCAM score, determining dose and lipophilicity in combination (or RO2) greatly improved the coherence among the different datasets, as shown in Figure 2.

DILI negative annotation are even more problematic, as demonstrated by a recent report [17] which suggested that as many as 40% of drugs defined as negatives by some means were also reported as DILI positives in other registries with established causality. Such contradictions, together with the significant variation in our assessment of risk factors, highlight the importance of selecting appropriately annotated datasets for the identification of DILI risk factors.

We therefore established a consensus annotation by cataloging the majority vote of the five selected annotations. Based on this consensus annotation, both lipophilicity and the extent of metabolism were significantly associated with increased DILI risk, and the strength of the association increased when combined with daily dose. The association between DILI risk and lipophilicity or metabolism was also consistently significant when assessed by the Chen and Greene annotations. In addition to annotation accuracy, an appropriate data analysis is crucial to ascertain correct conclusions. In a recent article, Weng et al. [29] reported that the combination of daily dose and metabolism or log*P* did not improve the prediction of drug-induced liver injury (DILI). Unfortunately, the data analysis is flawed, particularly when considering the methods for defining DILI negatives. Here, the authors made the assumption that DILI negatives could be defined by simply subtracting the positives for a given condition from the total number of drugs. In our reanalysis, however, we removed inappropriately assigned DILI negative drugs (e.g., “all human Adverse Drug Reactions (hADRs)” or “severe hADRs” in the study by Weng et al.), and found that the odd ratios for the combined daily dose and metabolism or log*P* were statistically significant, a finding distorted by the inappropriate assignment of DILI negatives in the study by Weng et al. [29]. This highlights the importance of a careful assessment of DILI annotations and the necessity of the consensus approach as employed in the present study. 

The mechanisms by which DILI occurs are complex and are frequently idiosyncratic in nature; nonetheless, lipophilicity and metabolism are contributing factors. Lipophilic drugs often display high volumes of distribution and are therefore distributed amongst many different tissues and organs and need to be converted into hydrophilic metabolites to be eliminated [23,30]. Furthermore, extensively metabolized drugs have a greater potential to form toxic reactive metabolites [15]. Toxic reactive metabolites may irreversibly form covalent bonds, inhibiting transport proteins or triggering an immune response. Reactive metabolites may also interfere with mitochondrial function or cause oxidative stress. Whether or not these interactions lead to liver injury appears to be dependent on the accumulation of the reactive metabolites beyond a critical level [2,31]. Hepatic exposure and the amount of parent drug or metabolite that accumulates depend on dose, which might explain why the combination of daily dose significantly improves the association between DILI risk and lipophilicity and metabolism.

Several limitations must be considered in this study. First, the lipophilicity and metabolism data used in this study were collected from different literature sources, yielding data that were likely not consistently measured or that may have been derived from different study protocols. Second, defining DILI risk associated with a drug is challenging and, without a “golden standard” annotation, relies on a variety of different sources and methods to collect data and define DILI risk, resulting in DILI annotations that differ for certain drugs. Third, our study is based on setting thresholds for daily dose and log*P* which could lead to some bias, especially for the drugs with values close to the thresholds. Fourth, not all of the datasets in the present study considered the causality assessment (e.g., RUCAM score), which is essential in future studies to characterize DILI, as reports suggest that some hepatotoxicity recorded in the literature is vague [27,28]. Finally, this study is a retrospective analysis and requires further validation based on a prospective study design.

Despite these limitations, our evaluation permitted the evaluation of five independently annotated datasets to overwhelmingly suggest that both lipophilicity and the extent of metabolism can be considered as risk factors of DILI. However, neither lipophilicity nor the extent of metabolism alone is a strong predictor for DILI risk, but together with daily dose, the association is clear.

## 4. Materials and Methods

### 4.1. Drug Datasets

To test the relationship between DILI risk, lipophilicity, and the extent of hepatic metabolism, we collected information for drugs included in the DILI rank dataset, which is a large dataset of 1036 drugs approved by the US FDA before 2010 [17].

The characterization of drugs according to their potential to cause liver injury was collected from the dataset by Chen et al., as shown in the Table S1 [17]. Chen et al. [17] systematically divided drugs into four categories (^v^Most-, ^v^Less-, ^v^No-DILI-concern, and Ambiguous DILI concern) based on FDA labeling and causality evidence determined by the Roussel Uclaf Causality Assessment Method (RUCAM) [24] or expert evaluation [25]. As in our previous study [16], only ^v^Most- and ^v^No-DILI-concern drugs were considered in detail. The ^v^Most-DILI-concern group includes drugs that, due to their potential to cause DILI, were withdrawn or given a boxed warning or severe DILI indication in their Warnings and Precautions. Included in the ^v^No-DILI-concern group are drugs with neither DILI indications in their labeling nor causality evidence.

Additional information for the 1036 drugs in the DILIrank dataset were collected from four publically available datasets in order to comprehensively assess the relationship between DILI risk, lipophilicity, and the extent of hepatic metabolism. The annotation by Greene et al. [19] classifies drugs based on human and animal evidence of toxicity, and only drugs categorized as having human evidence of toxicity (HH) or no evidence of human toxicity (NE) were used for this analysis. The annotation by Xu et al. [22] defines DILI positive drugs as those that, due to reports of hepatotoxicity, were withdrawn from the market or given warnings in their labeling, or those that had more than 10 clinical reports of serious hepatotoxicity. Those that not met the criteria of positives were defined as DILI negatives. In the annotation by Sakatis et al. [21], drugs were categorized as hepatotoxic or non-hepatotoxic based on the availability of clinical hepatotoxicity reported in literature and drug labeling. The annotation by Zhu et al. [20] classifies drugs as known hepatotoxicants, according to the assignment from Suzuki et al. [32], the causality of which has been judged by the RUCAM method. Conversely, drugs marketed for at least five years without reports of hepatotoxicity in PubMed or MedWatch were assigned as non-hepatotoxicants. All data were collected from original publications without further judgment on causality.

Additionally, we created a consensus annotation from the annotations by Chen [17], Greene [19], Zhu [20], Sakatis [21], and Xu [22]. In the consensus annotation, a drug will be considered as DILI positive if the majority of available annotations are positive, otherwise it will be considered as negative. Drugs with an equal number of available positive and negative annotations will be considered as positive because, in the annotations, DILI positives were justified with clinic evidences whereas DILI negatives were instead defined by a lack of evidence.

### 4.2. Daily Dose, Lipophilicity, and Metabolism

Daily dose for a total of *n* = 763 DILIrank drugs could be retrieved from the World Health Organization (WHO) Anatomical Therapeutic Chemical (ATC) database (http://www.whocc.no/atc_ddd_index) and from FDA-approved drug labels (http://dailymed.nlm.nih.gov/dailymed/about.cfm) and literature sources as described in our previous publication [16]. Log*P* values for *n* = 944 drugs were also collected, including *n* = 734 drugs of experimental log*P* values from the Drugbank database (www.drugbank.ca) and *n* = 210 drugs with calculated log*P* values from AlogPS 2.1 (http://www.vcclab.org/lab/alogps/start.html). 

A total of *n* = 640 DILIrank drugs were categorized as having significant or insignificant hepatic metabolism based on metabolism data collected from Micromedex^®^ 2.0 (http://www.micromedexsolutions.com/micromedex2/librarian/), NIH LiverTox database (https://livertox.nih.gov/), Lammert et al. [15], Drugbank [33], Pharmapendium (https://www.pharmapendium.com/), and literature sources. Significant metabolism was defined as ≥50%, as suggested by Lammert et al. [15]. For a small number of compounds (i.e., *n* = 16), no detailed information on the extent of metabolism was available; however, the amount of unchanged drugs in urine was <20% to suggest extensive metabolism. 

### 4.3. Statistical Analysis

The odds ratio with a 95% confidence interval derived from logistic regression was used to measure the strength of association between DILI risk and a specific risk factor (e.g., log*P* ≥ 3). A two-sided Fisher’s exact test was used to determine the statistical significance of the association. The logistic regression was computed using R software (version 3.2.2, R Development Core Team, Vienna, Austria) and the Bioconductor package (version 3.4, http://www.bioconductor.org/).

## Figures and Tables

**Table 1:** The assessment of the relationship between DILI risk, lipophilicity, and daily dose.

Annotated Datasets	DILI Classification	Positives	Negatives	Odds Ratio (95% Confidence Interval)	*p*-Value
Log*P* ≥ 3 and Daily Dose ≥ 100 mg					
Chen [17]	^v^Most-concern (*n* = 172)	71	101	11.50 (5.42–24.82)	<0.05
	^v^No-concern (*n* = 173)	10	163		
Greene [19]	Human hepatotoxicity (*n* = 174)	51	123	4.08 (1.57–11.23)	<0.05
	No evidence (*n* = 65)	6	59		
Zhu [20]	Hepatotoxic (*n* = 152)	42	110	5.47 (1.52–23.42)	<0.05
	Non-hepatotoxic (*n* = 46)	3	43		
Sakatis [21]	Hepatotoxic (*n* = 89)	28	61	2.80 (1.20–6.57)	<0.05
	Non-hepatotoxic (*n* = 78)	11	67		
Xu [22]	Positive (*n* = 179)	56	123	2.32 (1.27–4.24)	<0.05
	Negative (*n* = 128)	21	107		
Consensus	Positive (*n* = 313)	99	214	4.77 (2.79–7.86)	<0.05
	Negative (*n* = 255)	23	232		
Log*P* ≥ 3					
Chen [17]	^v^Most-concern (*n* = 172)	87	85	2.26 (1.42–3.58)	<0.05
	^v^No-concern (*n* = 173)	54	119		
Greene [19]	Human hepatotoxicity (*n* = 174)	69	105	1.72 (0.88–3.36)	0.098
	No evidence (*n* = 65)	18	47		
Zhu [20]	Hepatotoxic (*n* = 152)	64	88	2.31 (1.03–5.26)	<0.05
	Non-hepatotoxic (*n* = 46)	11	35		
Sakatis [21]	Hepatotoxic (*n* = 89)	35	54	1.03 (0.53–2.03)	1.0
	Non-hepatotoxic (*n* = 78)	30	48		
Xu [22]	Positive (*n* = 179)	74	105	0.97 (0.59–1.57)	0.91
	Negative (*n* = 128)	54	74		
Consensus	Positive (*n* = 313)	138	175	1.55 (1.09–2.22)	<0.05
	Negative (*n* = 255)	86	169		
Daily Dose ≥ 100 mg					
Chen [17]	^v^Most-concern (*n* = 172)	139	33	6.20 (3.71–10.39)	<0.05
	^v^No-concern (*n* = 173)	70	103		
Greene [19]	Human hepatotoxicity (*n* = 174)	123	51	2.65 (1.41–4.96)	<0.05
	No evidence (*n* = 65)	31	34		
Zhu [20]	Hepatotoxic (*n* = 152)	110	42	2.86 (1.37–5.96)	<0.05
	Non-hepatotoxic (*n* = 46)	22	24		
Sakatis [21]	Hepatotoxic (*n* = 89)	73	16	7.3 (3.41–15.83)	<0.05
	Non-hepatotoxic (*n* = 78)	30	48		
Xu [22]	Positive (*n* = 179)	127	52	2.52 (1.52–4.16)	<0.05
	Negative (*n* = 128)	63	65		
Consensus	Positive (*n* = 313)	225	88	3.54 (2.46–5.10)	<0.05
	Negative (*n* = 255)	107	148		

**Table 2:** The assessment of the relationship between DILI risk, the extent of metabolism, and daily dose.

Annotated Datasets	DILI Classification	Positives	Negatives	Odds Ratio (95% Confidence Interval)	*p*-Value
Hepatic Metabolism ≥ 50% and Daily Dose ≥ 100 mg					
Chen [17]	^v^Most-concern (*n* = 107)	76	31	11.09 (5.54–22.48)	<0.05
	^v^No-concern (*n* = 105)	19	86		
Greene [19]	Human hepatotoxicity (*n* = 139)	81	58	4.32 (1.91–9.92)	<0.05
	No evidence (*n* = 45)	11	34		
Zhu [20]	Hepatotoxic (*n* = 127)	70	57	5.32 (1.90–15.57)	<0.05
	Non-hepatotoxic (*n* = 32)	6	26		
Sakatis [21]	Hepatotoxic (*n* = 73)	53	20	7.48 (3.30–17.20)	<0.05
	Non-hepatotoxic (*n* = 65)	17	48		
Xu [22]	Positive (*n* = 141)	84	57	3.79 (2.11–6.84)	<0.05
	Negative (*n* = 100)	28	72		
Consensus	Positive (*n* = 221)	127	94	5.48 (3.40–8.84)	<0.05
	Negative (*n* = 177)	35	142		
Hepatic Metabolism ≥ 50%					
Chen [17]	^v^Most-concern (*n* = 107)	91	16	2.67 (1.27–5.40)	<0.05
	^v^No-concern (*n* = 105)	72	33		
Greene [19]	Human hepatotoxicity (*n* = 139)	109	30	2.42 (1.11–5.29)	<0.05
	No evidence (*n* = 45)	27	18		
Zhu [20]	Hepatotoxic (*n* = 127)	98	29	1.77 (0.70–4.42)	0.18
	Non-hepatotoxic (*n* = 32)	21	11		
Sakatis [21]	Hepatotoxic (*n* = 73)	61	12	1.80 (0.73–4.48)	0.21
	Non-hepatotoxic (*n* = 65)	48	17		
Xu [22]	Positive (*n* = 141)	112	29	1.92 (1.02–3.56)	<0.05
	Negative (*n* = 100)	67	33		
Consensus	Positive (*n* = 221)	174	47	1.90 (1.18–3.05)	<0.05
	Negative (*n* = 177)	117	60		
Daily Dose ≥ 100 mg					
Chen [17]	^v^Most-concern (*n* = 107)	87	20	7.67 (3.92–15.15)	<0.05
	^v^No-concern (*n* = 105)	38	67		
Greene [23]	Human hepatotoxicity (*n* = 139)	99	40	2.37 (1.12–5.00)	<0.05
	No evidence (*n* = 45)	23	22		
Zhu [20]	Hepatotoxic (*n* = 127)	90	37	3.13 (1.32–7.49)	<0.05
	Non-hepatotoxic (*n* = 32)	14	18		
Sakatis [21]	Hepatotoxic (*n* = 73)	61	12	7.63 (3.23–18.33)	<0.05
	Non-hepatotoxic (*n* = 65)	26	39		
Xu [22]	Positive (*n* = 141)	101	40	2.43 (1.37–4.30)	<0.05
	Negative (*n* = 100)	51	49		
Consensus	Positive (*n* = 221)	160	61	4.01 (2.57–6.26)	<0.05
	Negative (*n* = 177)	70	107		

## Supplementary Materials

Supplementary materials can be found at www.mdpi.com/1422-0067/18/7/1335/s1.
